# A Research on Low Modulus Distributed Fiber Optical Sensor for Pavement Material Strain Monitoring

**DOI:** 10.3390/s17102386

**Published:** 2017-10-19

**Authors:** Lingjian Meng, Linbing Wang, Yue Hou, Guannan Yan

**Affiliations:** National Center for Materials Service Safety, University of Science and Technology Beijing, Beijing 100083, China; b20150405@xs.ustb.edu.cn (L.M.); yhou@ustb.edu.cn (Y.H.); b20150406@xs.ustb.edu.cn (G.Y.)

**Keywords:** distributed optical fiber sensor, low modulus, asphalt concrete, encapsulation

## Abstract

The accumulated irreversible deformation in pavement under repeated vehicle loadings will cause fatigue failure of asphalt concrete. It is necessary to monitor the mechanical response of pavement under load by using sensors. Previous studies have limitations in modulus accommodation between the sensor and asphalt pavement, and it is difficult to achieve the distributed monitoring goal. To solve these problems, a new type of low modulus distributed optical fiber sensor (DOFS) for asphalt pavement strain monitoring is fabricated. Laboratory experiments have proved the applicability and accuracy of the newly-designed sensor. This paper presents the results of the development.

## 1. Introduction

The state of the strain and stress distribution of pavement structure/material is critical to the health state of the road, where lots of research has been conducted [1,2,3]. In order to monitor the strain distribution of the pavement structure and pavement material under actual loads, sensors are very important. In order to obtain a complete strain distribution of the pavement structure, a large number of sensors are usually required. Additionally, several special attributes are also needed for pavement sensors such as high-temperature resistance and corrosion resistance.

Optical fiber sensing technology has been studied a great deal. Compared with traditional electrical sensors, optical fiber sensors have many advantages, such as being small in size, lightweight, and resistant to anti-electromagnetic interference and corrosion. Moreover, in an optical fiber sensing system, the optical fiber is not only a sensor, but also a transmission medium. It can achieve distributed sensing [4].

In recent years, many researchers have used optical fiber sensors for pavement health monitoring [5,6,7,8]. These sensing technologies mainly use a quartz optical fiber as the sensing element, and this type of optical fiber is very fragile; the ultimate tensile strain is about 1% [9]. If the sensor has no protection, it is easy to damage it during the pavement spreading and installation process, which leads to a low survival rate.

In order to improve the survival rate of the optical fiber sensor, much work has been done on encapsulation methods for optical fiber sensors. Zhou et al. conducted a study on the strain transfer characteristics of fiber Bragg grating (FBG) sensors with different adhesives and bounded length. The strain transfer rate can be improved by either increasing the bonded length, the thickness of the adhesive, or choosing the proper adhesive [10]. Zhang et al. developed a new type of distributed optical fiber sensor. The optical fiber sensor is pasted on metal banding. This method improves the strength of the sensor significantly [11]. Zhou et al. developed a steel capillary encapsulating technique for FBG sensors. The temperature and strain sensing properties of the encapsulated FBG sensor were experimentally studied [12]. Inaudi et al. developed an adequate packaging of the sensors to monitoring cracks in concrete structures [13].

However, there are still some problems in the application of fiber optic sensors in pavement strain monitoring. The modulus of encapsulation structure for optical fiber sensor is too large compared with asphalt concrete. This will affect the original strain field of the pavement structure. Additionally, a quasi-distributed sensing technique, such as FBG, is difficult to monitor the complete strain distribution of pavement. Therefore, it is important to develop a new type of fiber optic sensor with a low-modulus encapsulation structure to achieve distributed monitoring of the pavement.

In this paper, a new type of low-modulus encapsulation structure for distributed optical fiber sensors (DOFS) is designed and fabricated. The main research contents of this paper are as follows:

1. A silicon rubber strip encapsulation structure for DOFS is designed.
2. Based on finite element analysis, the influence of the encapsulation structure on strain monitoring is studied, and the encapsulation method is optimized.
3. The performance of the DOFS with rubber encapsulation is measured through laboratory experiments.

## 2. Packaging of the Optical Fiber Sensor

### 2.1. Operation Principle

The distributed optical fiber sensing was conducted with LUNA Innovations Co., Ltd. (Roanoke, VA, USA) ODISL-B optical distributed sensor interrogator. This device can measure the distributed strain along a single-mode optical fiber with a refresh rate up to 50 Hz. The strain measurement range is ± 10,000 με, and the maximum measuring range is 20 m. The spatial resolution is 2.6 mm. We use 62.5/125 μm single-mode optical fiber produced by Corning Incorporated (NYC, NY, USA).

**Figure 1:**
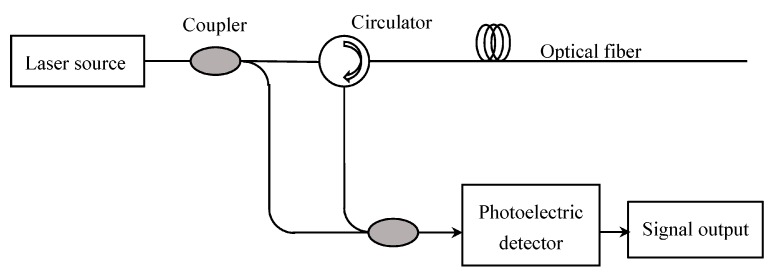
Operation principle of the OFDR system.

**Figure 2:**
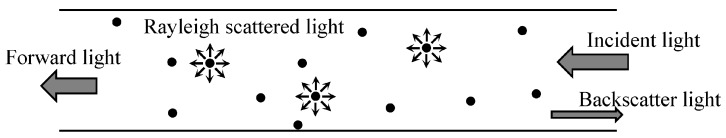
Schematic illustration of Rayleigh scattering in an optical fiber.

The operation principle of this device is OFDR (optical frequency domain reflectometry). The structure of the OFDR system is shown in Figure 1 and Figure 2 [14]. 

The device injects a light into the optical fiber, and the energy of the optical pulse will be continuously lost in the transmission process due to the light absorption of the optical fiber material and backscatter characteristics of the optical fiber. Rayleigh scattered light is a scattered light which has the same frequency as the incident light. In general, the spectral response of Rayleigh scattering is mainly affected by the strain and temperature. The change of Rayleigh scattering can be calibrated, and converted to show the changes in temperature and strain as follows. First, we measure the Rayleigh scattering data of any region of the fiber in specified environment as a reference value; second, we measured the data of Rayleigh scattering at the targeted region. By comparing the difference between the reference value and the measured value, the information of strain or temperature change can be obtained.

The spectral shift caused by strain and temperature variation can be given as Equation (1) [15]:

(1) $$\frac{\Delta \lambda }{\lambda }=-\frac{\Delta \nu }{\nu }=K_{t}\Delta t+K_{s}\varepsilon$$

where λ and ν are the average wavelength and frequency, respectively. $K_{t}$ and $K_{s}$ are the temperature and strain calibration constants. The typical values of the constants as:

(2) $$K_{t}=6.45\times 10^{-6}\;℃$$

(3) $$K_{s}=0.78$$

### 2.2. Encapsulation Structure Design

The elastic modulus of asphalt pavement (1000 Mpa) [16] requires that the modulus of the sensor cannot be too large. In the previous study, a variety of methods have been researched to improve the strain transfer rate of the optical fiber sensor for pavement strain monitoring. Wang et al. used two grid blocks to fix both ends of the FBG sensor. Strain sensing is performed by elongating the distance between two grid blocks [17]. Zhou et al. combine FBG sensors with a stainless annulus and FRP (fiber reinforced polymer). Laboratory experiments and finite element analysis have proved the applicability of the sensor [18]. However, some problems still exist in the encapsulation structure. Theoretical calculation and engineering application are quite different. The perfect interaction condition between the asphalt concrete and the sensor package are difficult to achieve due to the uneven aggregation and differences in construction skills. Additionally, these methods are only applicable for quasi-distributed strain monitoring, and cannot be applied to distributed measurements.

**Figure 3:**
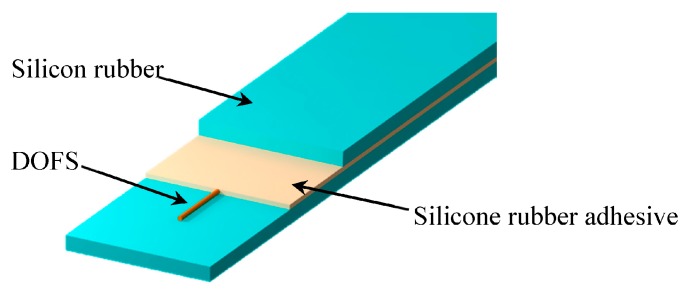
Schematic illustration of DOFS with the silicone rubber package.

Given the analysis above, a new type of encapsulation structure for DOFS is put forward in this article. Laboratory experiments have proved the applicability and accuracy of the newly-designed sensor. The packaging structure of DOFS is shown in Figure 3.

### 2.3. Strain Actuation Analysis

In this study, silicone rubber, produced by Shanghai Shuangcheng Plastic Material Co., Ltd. (Shanghai, China), was chosen as the primary encapsulating material. This kind of rubber can withstand −55 °C to 180 °C without losing elasticity performance. It can meet the temperature requirements of pavement monitoring.

**Figure 4:**
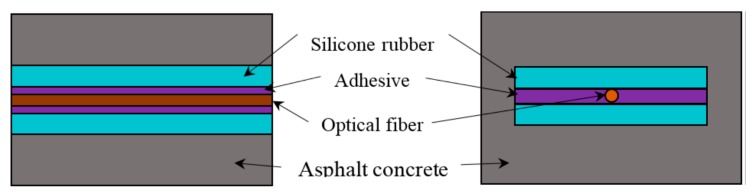
Cube model of the DOFS with a rubber encapsulation structure.

**Figure 5:**
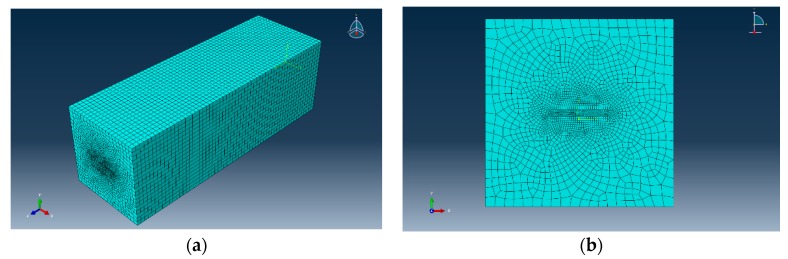
Mesh model for FEA calculation. (**a**) Isometric view of the mesh model; (**b**) front view of the mesh model.

In order to investigate the strain actuation rate of rubber encapsulation structure, the software ABAQUS is used to create a model of the DOFS with the silicone rubber package and to calculate the strain distribution of the optical fiber. The cube model of DOFS with rubber encapsulation structure is shown in Figure 4. The mesh model of FEA is shown in Figure 5. It is assumed that every layer is made of isotropic material with linear elastic mechanical behavior. The material parameters of the FEA model are referred to [19,20]. An axial displacement of 0.003 m is applied to the ends of the asphalt concrete.

**Figure 6:**
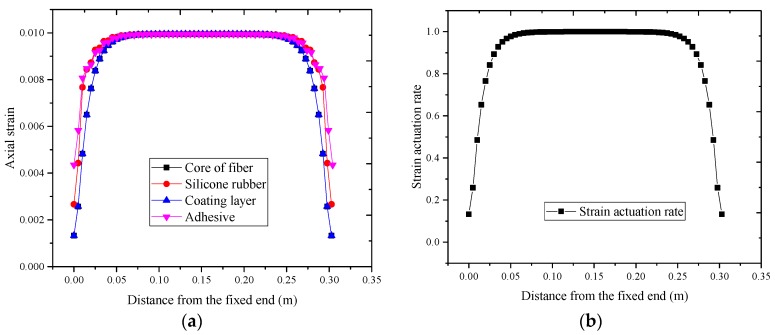
(**a**) Axial strain distribution of each layer; and (**b**) strain actuation rate of DOFS.

Axial strain located at the symmetry surface of each layer is shown in Figure 6a. As shown in Figure 5 that the axial strain of each layer is non-uniform along the bonded length. Due to the strain concentration of each layer at the bonded end region, the strain of each layer rapidly decreases with increasing distance between the bonded end region and the mid-beam region. Additionally, the strain of each layer at the mid-beam region is approximately equal. As shown in Figure 6b, the actuation rate of the optical fiber gradually increases from the bonded end region to the mid-beam region of the bonded length, and it is close to 1 at the mid-beam region.

### 2.4. Fabricating Process

**Figure 7:**
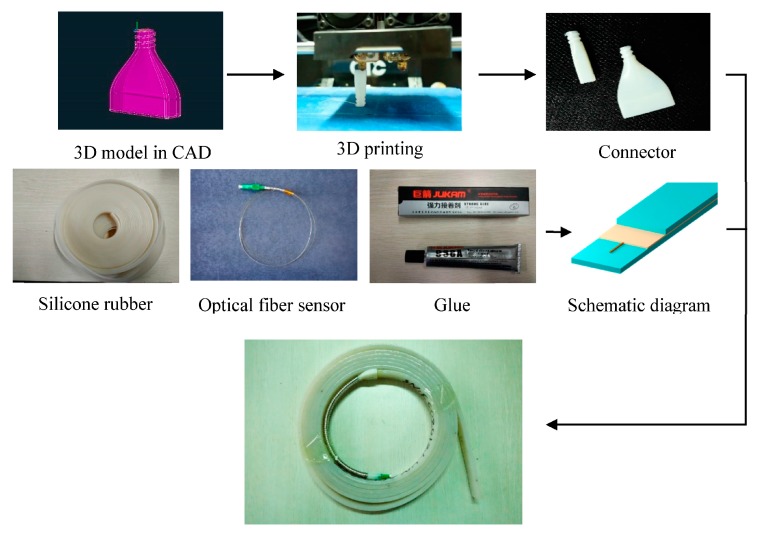
Fabricating process.

**Figure 8:**
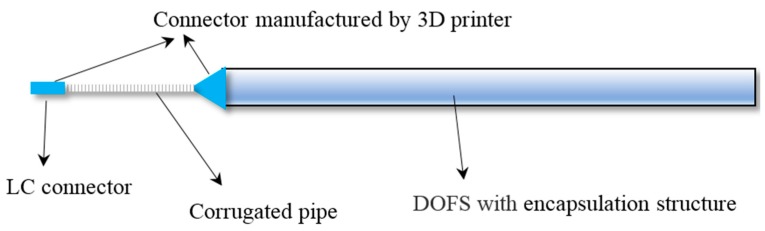
Schematic illustration of DOFS with the encapsulation structure.

The fabricating process of the DOFS with the encapsulation structure includes the following six steps (Figure 7): (1) Two connectors are designed by CAD software and manufactured by a 3D printer. (2) After cleaning and drying, a positioning line in the middle of the rubber strip is drawn. (3) The components are combined together, as shown in Figure 8. (4) The optical fiber is temporarily fixed using instant adhesive. (5) Special silicone adhesive named JUKAM-988A, produced by Dongguan Jingshun compound material Co., Ltd. (Dongguan, China), has been used to paste two pieces of silicone rubber together. (6) It takes 24 h for the adhesive to dry. After that, the DOFS with the encapsulation structure needs to be placed in an oven at 70 °C for 6 h.

## 3. Sensing Performance Test

### 3.1. Interface Examination of Rubber Encapsulation Structure

In order to observe the interface contact of the encapsulation structure, three sliced specimens of DOFS with rubber encapsulation structures have been prepared and observed with a scanning electron microscope (ZEISS EVO18, ZEISS Co., Oberkochen, Germany) at different standardized magnifications (200×, 500×, and 2000×).

**Figure 9:**
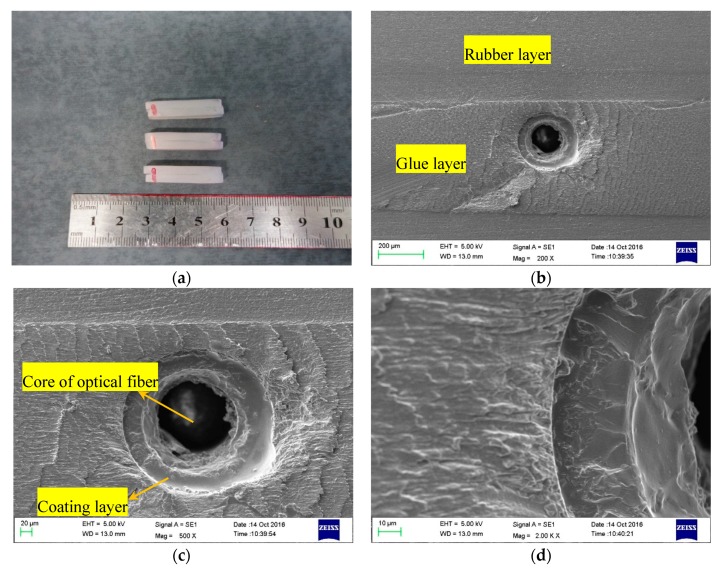
(**a**) Three sliced specimens after polishing and cleaning; (**b**) scanning electron microscope (SEM) image of sample No. 3 (200×); (**c**) SEM image (500×); and (**d**) SEM image (2000×).

As shown in Figure 9, the interfaces between different layers adhered tightly. The thickness of the adhesive is about 500 μm.

### 3.2. Tensile Sensing Test

**Figure 10:**
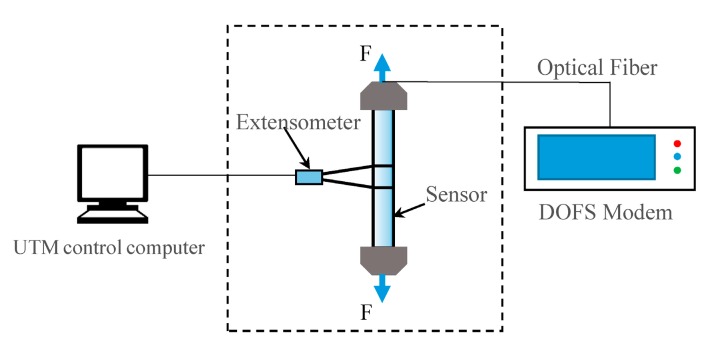
Schematic illustration of tensile sensing test.

**Figure 11:**
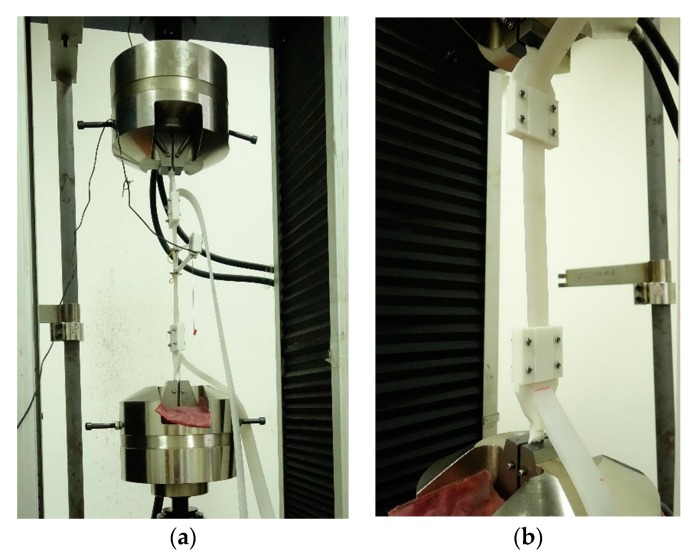
(**a**) The sensor is under testing; and (**b**) the connection structure between the sensor and the UTM.

During the process of optical fiber sensing test, a 5 m long sensor is fabricated and tested. Five test sections are chosen evenly along the sensor. The tensile sensing test was carried out with a Jinan Koohei Test Machine Co., Ltd. (Jinan, China) WDW-200D universal test machine (UTM). The displacement measurement accuracy is ±1% with a displacement resolution up to 0.05 mm. An extensometer is used to measure the tensile strain of the sensor. The schematic illustration of the tensile sensing test is shown in Figure 10, and the test is shown in Figure 11.

In the test process, the stretching length of the sensor is controlled by the extensometer. The sampling rate of the DOFS modem is set to 50 Hz and the spatial resolution is set to 2.6 mm. In order to protect the sensor without being pulled off, the strain of the sensor is limited to less than 13,000 με. 

**Figure 12:**
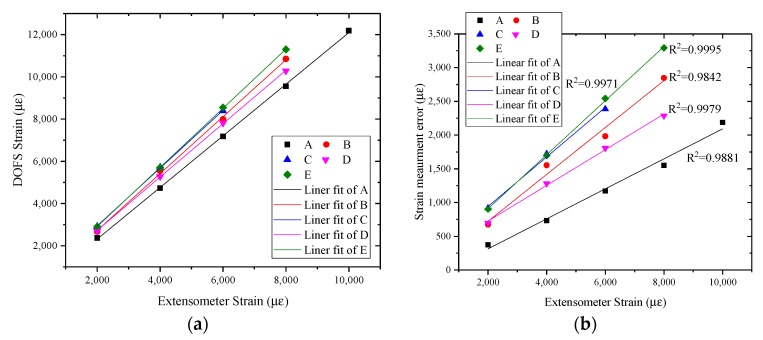
(**a**) Strain data of tensile sensing test; and (**b**) strain measurement error.

As shown in Figure 12a, the strain measurement data from different test sections of the optical fiber sensor shows great linearity, the $R^{2}$ of each linear fit function is greater than 0.99. 

During the test, when replacing another test section of the sensor, the extensometer needs to be reinstalled. The difference of the strain measurement data between different test sections were found in the data output due to the limitation of the extensometer installation’s stability.

The strain measurement error is defined as follows:

(4) $$\Delta \varepsilon =\varepsilon_{f}-\varepsilon_{e}$$

where Δε is the strain measurement error; $\varepsilon_{f}$ is the measured value of optical fiber sensor; and $\varepsilon_{e}$ is the measured value of extensometer.

As shown in Figure 12b, the strain measurement error gradually increases with the increase of the measured value of the extensometer. The strong correlation between strain measurement error and measured value of the extensometer of different test section shows that the unstable connection between the extensometer and test sections of optical fiber sensors will cause strain measurement errors.

### 3.3. Compression Test of Concrete Column

**Figure 13:**
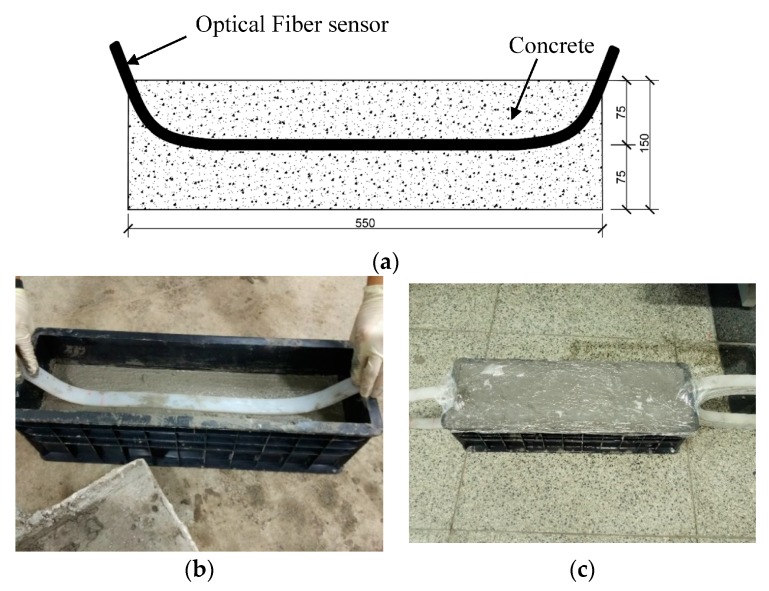
The concrete column made for test. (**a**) Schematic illustration of the concrete column (mm); (**b**) production process; (**c**) concrete curing.

In this test, a cement concrete column was made, the size of the column is 550 mm × 150 mm × 150 mm. The optical fiber sensor is placed in the center of the column, as shown in Figure 13. The concrete mix ratio is shown in Table 1.

**Figure 14:**
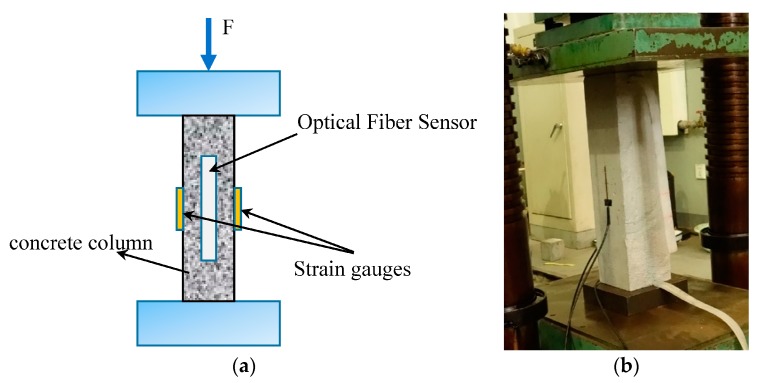
(**a**) Schematic illustration of the compression test; and (**b**) the concrete column under testing.

After 28 days of curing, three 10 cm long strain gauges are pasted onto the surface of the concrete column. The strain of the resistance strain gauge is measured by a static strain tester (Donghua DH3818, Donghua Test Co., Ltd., Taizhou, China) (Figure 14).

**Figure 15:**
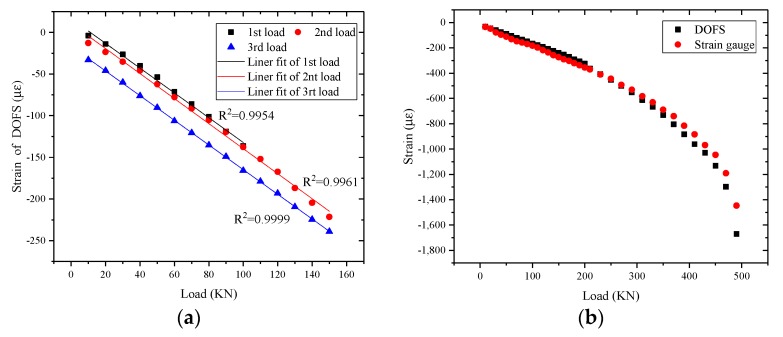

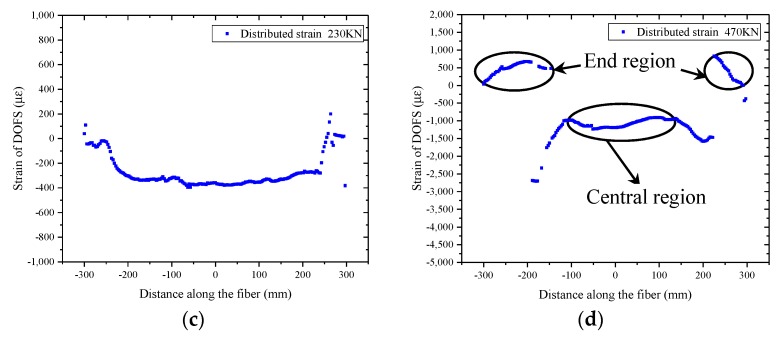
(**a**) Load-strain curve of three load-unload cycle; (**b**) load-strain curve from 0 KN to 490 KN; (**c**) distributed strain of the concrete column when the load is 230 KN; and (**d**) the distributed strain of the concrete column when the load is 470 KN.

**Figure 16:**
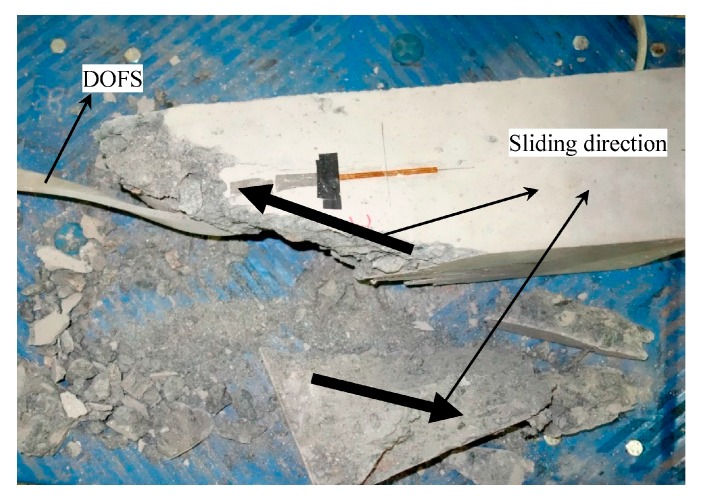
The concrete column after failure.

As shown in Figure 15a, the strain measurement data from different load cycles of the concrete column possesses good repeatability and linearity. The plastic deformation of the concrete column between different load-unload cycles is also shown in this figure. It can be verified that the stickiness between the encapsulation structure, the concrete, and the optical fiber are all reliable. Due to the crack developing, the strain measured by strain gauges is a slightly smaller when the load is larger than 200 KN (Figure 15b). The strain measured by strain gauges will not increases rapidly until the crack has passed through the strain gauge. The optical fiber sensor can measure the strain distribution along the fiber, so there is a greater opportunity to monitor the development of cracks. Therefore, the strain measured by the DOFS grows faster than the strain measured by strain gauges. As shown in Figure 15c, the optical fiber sensor measures the strain distribution along the fiber. Since the direction of the optical fiber is not parallel to the direction of the axial strain, the strain measurement value of the optical fiber gradually decreases from the central region to the end region, and it is close to 0 at the end region. As shown in Figure 15d, strain increases rapidly near the end region of the concrete column. Many longitudinal cracks can be found at both ends (Figure 16). The optical fiber sensor which is close to the end region has been tensioned and the strain measurement value is positive.

### 3.4. Bending Test of Asphalt Concrete Beam

In this test, an asphalt concrete beam is made. The size of the beam is 100 mm × 100 mm × 400 mm. The DOFS with encapsulation structure is placed at the bottom of the beam. The bending test was carried out with a UTM and a four-point bending fixture. The component of asphalt mixture is shown in Table 2.

**Figure 17:**
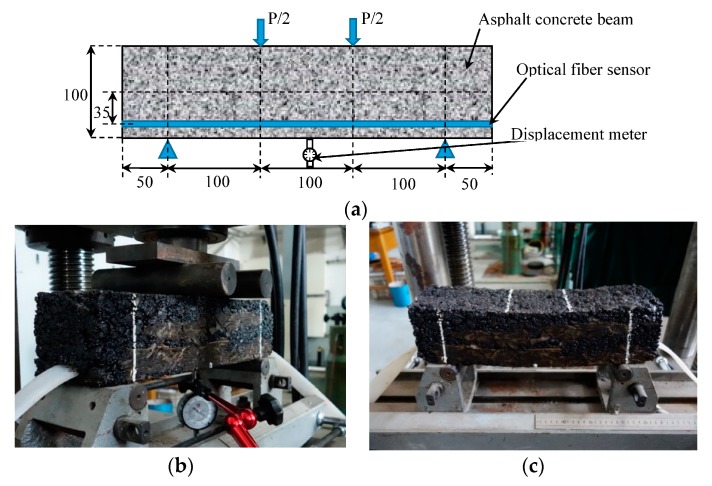
Four-point bending test of an asphalt concrete beam. (**a**) Schematic illustration of the asphalt concrete beam (mm); (**b**) testing process; (**c**) asphalt concrete beam after bending test.

The load increases from 0.5 KN to 3.5 KN and the loading is increased 0.5 KN per step. A displacement meter is placed at the bottom of the beam to measure the bending deflections (Figure 17). After each loading, keep the load and read the value of the displacement meter. Then unload. The final fracture loading of the asphalt concrete beam is 3.46 KN.

According to the principle of material mechanics, the strain of the optical fiber sensor can be obtained by the following formula [21]:

(5) $$\varepsilon_{s}=\varepsilon_{b}\times \frac{y}{\left(\frac{h}{2}\right)}=\frac{180\times t\times h}{23\times L^{2}}\times \frac{2\times y}{h}$$

Where $\varepsilon_{s}$ is the strain measured by DOFS; $\varepsilon_{b}$ is the strain at the bottom of the beam; *y* = 35 mm; *h* = 100 mm; *t* is the deformation of the mid-beam region of the asphalt concrete beam measured by the displacement meter; and *L* is the distance between the two lower fulcrums. 

**Figure 18:**
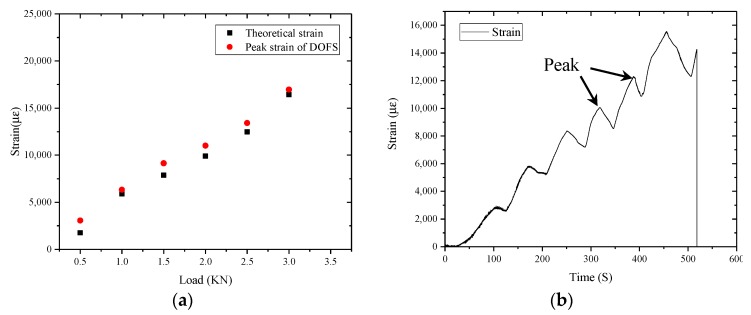
(**a**) The theoretical strain and strain measured by sensor; and (**b**) the change of the mid-span strain value.

As shown in Figure 18a, the strain of the mid-beam region of the beam gradually increase with load increase. The pack strain measured by DOFS is always larger than the theoretical strain based on bending deflections measured by the displacement meter and calculated by Equation (3). The reason is, in the measuring process of the displacement meter, the load of the universal testing machine should be kept constant. During this process, the strain of the beam continuously increased due to the creep properties of the asphalt concrete. 

As shown in Figure 18b, The DOFS record the entire process of loading. The creep process and unload process are also shown in this figure.

## 4. Conclusions

In this paper, a new type of low-modulus distributed optical fiber sensor (DOFS) for asphalt pavement strain monitoring is designed and fabricated. Silicone rubber is chosen as the encapsulation material of the optical fiber, which has good thermal stability and durability.

1. The SEM analysis and FEA results show that the key to making the strain measurement accurate is to ensure the effective bonding between the optical fiber sensor and the package structure.
2. The FEA results also show that the rubber encapsulated fiber optic sensors require a certain length of the strain actuation region to achieve the coordinated deformation with the asphalt concrete. The strain actuation rate of rubber encapsulation structure is acceptable for pavement engineering.
3. The compression test of a concrete column and bending test of an asphalt concrete beam verified that the rubber package structure can effectively protect the optical fiber sensor without damage by the vibration and extrusion during the fabricating and testing process.

The experimental results and the conclusions show that the distributed optical fiber sensor with a novel low-modulus rubber package structure can accurately measure the pavement material strain distribution. It is promising to develop a new optical fiber sensing system for pavement monitoring in the future.

## Figures and Tables

**Table 1:** The concrete mix ratio.

Component	Quality/kg
Water	1.346
Cement (P·O 42.5)	5.855
Sand	13.163
Gravel	14.742
Quick-setting agent	0.024

**Table 2:** Asphalt mixture mix ratio.

Aggregate	Quality/g
10–15 mm	4285.7
5–10 mm	3428.6
2.36–4.75 mm	1571.4
<2.36 mm	4285.7
Slag	714.3
Asphalt	714.3
